# Imaging Fibrogenesis in a Diet-Induced Model of Nonalcoholic Steatohepatitis (NASH)

**DOI:** 10.1155/2019/6298128

**Published:** 2019-12-01

**Authors:** S. V. Hartimath, R. Boominathan, V. Soh, P. Cheng, X. Deng, Y. C. Chong, F. F. Yong, P. W. Tan, W. Han, E. G. Robins, J. L. Goggi

**Affiliations:** ^1^Singapore Bioimaging Consortium, Agency for Science Technology and Research (A∗STAR), 11 Biopolis Way, #01-02 Helios, Singapore 138667, Singapore; ^2^Clinical Imaging Research Centre (A∗STAR–NUS), Yong Loo Lin School of Medicine, National University of Singapore, Singapore 117599, Singapore

## Abstract

**Purpose:**

Liver fibrosis is the hallmark of chronic nonalcoholic steatohepatitis (NASH) and is characterised by the excessive deposition of extracellular matrix proteins. Early detection and accurate staging of liver fibrosis is critically important for patient management. One of the earliest pathological markers in NASH is the activation of hepatic stellate cells (HSCs) which may be exploited as a marker of fibrogenesis. Activated HSCs secreting factors such as integrin *α*_v_*β*_3_ propagate fibrosis. The purpose of the current study was to assess the utility of the integrin *α*_v_*β*_3_ imaging agent [^18^F]FtRGD for the early detection of fibrosis in a diet-induced model of NASH longitudinally using PET imaging.

**Procedures:**

Mice were fed with either standard chow diet (SD), high-fat diet (HFD), or a choline-deficient, L-amino acid-defined high-fat fibrogenic diet (CDAHFD) to mimic the clinical pathology of liver disease and followed longitudinally for 10 weeks to assess the development of liver fibrosis using [^18^F]FtRGD positron emission tomography (PET) imaging. Standard blood biochemistry, histological measures, and qPCR were used to quantify integrin *α*_v_*β*_3_, smooth muscle actin, and collagen types 1 and 6 to assess the extent of NASH pathology and accurately stage liver fibrosis.

**Results:**

The CDAHFD fibrogenic diet predictably developed hepatic inflammation and steatosis over the 10 weeks studied with little NASH pathology detected in high fat diet-treated animals. Stage 1 fibrosis was detected early by histology at day 21 and progressed to stage 2 by day 35 and stage 3 by day 56 in mice fed with CDAHFD diet only. Noninvasive imaging with [^18^F]FtRGD correlated well with integrin *α*_v_*β*_3_ and was able to distinguish early mild stage 2 fibrosis in CDAHFD animals compared with standard chow diet-fed animals at day 35. When compared with high fat diet-fed animals, [^18^F]FtRGD was only able to distinguish later moderate stage 2 fibrosis in CDAHFD animals at day 49.

**Conclusions:**

The diet-induced progression of liver fibrosis was confirmed using histology and correlated well with the mRNA of integrin *α*_v_*β*_3_ and extracellular matrix protein expression. [^18^F]FtRGD showed very good correlation between liver uptake and integrin *α*_v_*β*_3_ expression and similar detection sensitivity to the current clinical gold standard modalities for staging of liver fibrosis.

## 1. Introduction

Non-alcoholic steatohepatitis (NASH) is a huge clinical burden and is estimated to increase substantially in the near future. Nearly 50% of NASH patients develop liver fibrosis, which, if not detected at an early stage, can lead to liver cancer or organ failure [1]. Biopsies are routinely used to diagnose liver fibrosis, but these are invasive, error-prone, and provide little information about tissue heterogeneity. Staging of liver fibrosis is critically important for appropriate diagnosis and patient management and has been shown to be the strongest independent risk factor for predicting transplantation requirement and mortality. Sensitive and accurate assessment of the fibrotic stage throughout the liver is therefore critical for prognosis, and early detection of fibrosis allows for interventions which can reverse the pathology. Fibrosis is characterized by the excessive deposition of extracellular matrix (ECM) components resulting from chronic tissue injury or inflammation [2]. Noninvasive imaging technologies have been assessed over the years for their ability to stage fibrosis and assess therapy response, but all strategies have limitations and significant improvements in sensitivity are needed. The most promising noninvasive imaging technologies include ultrasound- and MR-based elastography, while these imaging methodologies are useful in assessing liver stiffness; they provide little information on the molecular pathology involved [3, 4]. The pathology of NASH and the development of liver fibrosis have been well documented [2]. Activation of hepatic stellate cells (HSCs) is one of the earliest cellular pathologies in NASH and may be exploited as a marker of fibrogenesis. HSCs play a key role in fibrogenesis by secreting factors such as integrin *α*_v_*β*_3_ that propagate fibrosis. Integrin *α*_v_*β*_3_ plays a critical role in transforming myofibroblast cells to express *α*-smooth muscle actin (*α*-SMA) which leads to excessive production of ECM proteins such as collagen type-1 and type-6 [2]. Thus, radiopharmaceuticals that can monitor the expression of integrin *α*_v_*β*_3_ might be useful as early markers to detect fibrogenesis.

Integrin *α*_v_*β*_3_ binders (^125^I and ^99m^Tc-labeled cRGD) have previously been used to detect liver fibrosis in chemically induced animal models [5–11]. However, chemically induced NASH models develop fibrosis very rapidly and do not recapitulate the clinical progression of the disease. Furthermore, these models severely damage the hepatocytes, liver parenchyma, and Kupffer cells within days. In NASH pathology, inflammation occurs much more slowly and HSCs are activated at a very early stage of fibrosis. In the present study, we have investigated the relationship between the hepatic uptake of [^18^F]FtRGD, an integrin *α*_v_*β*_3_ selective probe, and the development of fibrosis using PET imaging in a diet-induced NASH model, the low methionine, choline-deficient high-fat diet (CDAHFD). The CDAHFD diet more faithfully reproduces clinical pathology from uninjured parenchyma to steatosis and NASH, where hepatic stress caused by fatty acid flux from adipose tissue to the liver as well as elevated storage of triglycerides leads to fibrosis and liver dysfunction [12–14].

## 2. Materials and Methods

### 2.1. Animals and Diet Intervention

Male C57/BL6 mice were purchased from InVivos (Singapore). C57/BL6 mice were chosen as a rodent strain as they have been shown to develop hepatic pathology with the chosen diet. Mice were fed either standard chow diet (*n* = 5), a high-fat diet (HFD), rodent purified diet w/60% energy from fat (HFD : 58Y1, *n* = 5) or a high fat fibrogenic diet (AA defined high-fat diet w/no choline and 0.1% methionine; CDAHFD with 60% energy from fat; 9 GKW was purchased from Test Diet, Richmond Indiana, USA, (*n* = 5) for ten weeks.

### 2.2. Biochemical Assays and Histopathological Evaluation

All animals were monitored and body weight was measured twice a week. Blood was collected from the orbital plexus, and plasma was separated (200 *μ*L) for triglyceride analysis (TG) (ab65336, Abcam). Animals were sacrificed, and the liver was collected at 21, 28, 42, 56, and 70 days of CDAHFD and HFD treatment. The liver was weighed, and a portion was snap frozen for hydroxyproline analysis (ab222941, Abcam) or mRNA quantification. The remaining liver tissues were fixed in 10% neutral buffered formalin for the histological examination.

Formalin-fixed tissues were sectioned (4 *μ*m) and stained with Picro Sirius Red (PSR) or hematoxylin-eosin (H&E). The liver specimens were microscopically evaluated for the presence of inflammatory cells, hepatocellular ballooning, fat changes (steatosis), fibrosis, and other lesions as per the grading practice previously described [12]. The pathological grading for the different stages of fibrosis is based on the severity and classified as follows: for inflammatory cell infiltration, grade 0: none; grade 1: 1-2 foci; grade 2: 3-4 foci; and grade 3: more than 4 foci observed at 200x. For hepatocellular ballooning and degeneration, grade 0: none; grade 1: few balloon cells; and grade 2: cells/prominent ballooning. For fatty changes (steatosis), grade 0, the absence of steatosis; grade 1, < 30% of hepatocytes affected; grade 2, 30–70% of hepatocytes affected; and grade 3, > 70% of hepatocytes affected. For hepatic fibrosis (based on PSR staining), stage 0: none; stage 1: mild, perisinusoidal, or periportal; stage 2: moderate, perisinusoidal, and periportal; stage 3: bridging fibrosis; and stage 4: cirrhosis.

### 2.3. Preparation of [^18^F]FtRGD

All chemicals and solvents obtained commercially were of analytical grade and used directly without further purification. [^18^F]FtRGD was prepared as a two-pot reaction as previously described [15] based on a modified method by Bejot et al. [16]. Briefly, the azeotropic dried [^18^F]fluoride was reacted with 2-azidoethyl-4-toluenesulfonate (2 *μ*L) in dry acetonitrile (0.5 mL) to afford 2-[^18^F]fluoroethyl azide ([^18^F]FEA) [17]. The conjugation of 2-[^18^F]fluoroethyl azide to alkyne-functionalized c(RGDyK) peptide via the Cu(I)-catalysed Huisgen 1,-3 dipolar cycloadditions. After the reaction, [^18^F]FtRGD was purified using semipreparative HPLC followed by solid-phase C18 light cartridge extraction and reformulated in phosphate buffered saline (pH = 7.4). The tracer was sterilized by passing through a 0.22 *μ*m Millex GV filter before use in animals. The radiochemical purity was >98%.

### 2.4. PET Imaging

All animals (*n* = 5 per group) were longitudinally imaged from 3 to 10 weeks post initiation of diet regime. The animals were injected with a solution of [^18^F]FtRGD (∼10 MBq in 0.2 ml) *via* the lateral tail vein. After 60 min after injection, animals were imaged using a Siemens Inveon PET-CT (Siemens, Germany) with a 10 min static followed by a standard CT scanning protocol as previously described [16]. PET data were acquired in the list mode, and images were generated from sinogram data, followed by 3-dimensional ordered subset expectation maximization (OSEM-3D) reconstruction and attenuation correction using CT. The PET and CT images were coregistered to confirm the anatomical location, and radiopharmaceutical uptake was determined by drawing a region of interest (ROIs) over the liver delineated using the CT images. The tissue concentrations were measured using ROI analysis in Amide software (Sourceforge 10.3, http://amide.sourceforge.net), and the uptake of the tracer are presented as a percentage change in liver uptake.

### 2.5. Immunohistochemical Analysis

Liver samples were snap frozen in liquid nitrogen and sectioned using a cryotome (5 *μ*m slices) for immunohistochemical examinations. The sections were soaked in methanol containing 0.3% H_2_O_2_ for 30 min at room temperature to fix and block endogenous peroxidase activity and then washed with PBS. After blocking with goat serum/H_2_O_2_, the tissues were incubated with primary antibody at room temperature for 30 minutes time (rabbit polyclonal antialpha SMA Abcam, ab15734; at 1 : 200 dilution). The sections were then processed for HRP-conjugated secondary antibody as per the manufacturer's protocol polymer (30 mL) Antirabbit Poly-HRP-IgG (<25 *μ*g/mL) containing 10% (v/v) animal serum in tris-buffered saline/0.09% ProClin™ 950 using diaminobenzidine (DAB) as a chromogen and counterstained with hematoxylin. The grading was carried out based on intensity of *α*-SMA expression (minimal; <25%, mild; 25–50%, moderate; 50–75%, and intense; >75%).

### 2.6. Gene Expression Analysis by qPCR

Liver tissue was snap frozen and processed for hepatic RNA extraction and subsequent qPCR analysis as previously described [18]. TRIzol (Thermo Fisher Scientific) extracted total RNA, and cDNA was amplified using the indicated primers by 40 cycles of PCR. The mRNA expression level of integrin *α*_V_*β*_3_, collagen type 1 and 6 alpha 1 was quantified by qPCR. The 2^−ΔΔCT^method was used to estimate the relative mRNA expression and normalized to 18S mRNA (as a housekeeping gene). Sequences of the primers used for qPCR are as follows: Col1a1: 5′-GCTCCTCTTAGGGGCCACT-3′ and 5′-CCACGTCTCACCATTGGGG-3′. Col6a1: 5′-CTGCTGCTACAAGCCTGCT-3′ and 5′-CCCCATAAGGTTTCAGCCTCA-3′. Integrin *α*_V_*β*_3_: 5′-CCGTGGACTTCTTCGAGCC-3′ and 5′-CTGTTGAATCAAACTCAATGGGC-3′. 18S: 5′-CGTGATTAGCGATGATGAACCAGG-3′ and 5′-CATCTCGAGCAA-GTCTTTCAGTCC-3′.

### 2.7. Statistical Analyses

All data are expressed as mean ± SD. Statistical significance was assessed using a 1-way analysis of variance (ANOVA) with Bonferroni post hoc test, and *p* < 0.05 was considered statistically significant. All the statistical analyses were performed using GraphPad Prism 8.0 (GraphPad Software, Inc. La Jolla, CA) and Microsoft Excel 2016.

## 3. Results

### 3.1. Physiological and Histopathological Liver Analysis

As shown in Table 1, CDAHFD mice showed significant increases in liver weight due to ectopic fat deposition and the accumulation of triglycerides and extracellular matrix proteins, consistent with the development of nonalcoholic fatty liver disease (NAFLD) [12] and NASH. HFD mice also showed some increase in liver weight, likely due to ectopic fat deposition, but no significant triglyceride or extracellular matrix protein accumulation. Serum analysis revealed a significant increase in triglycerides (TG) and hydroxyproline levels in CDAHFD-fed mice compared with HFD mice from day 35 (*p* < 0.035 and *p* < 0.045, respectively) which continued until the end of the assessment period.

**Figure 1:**
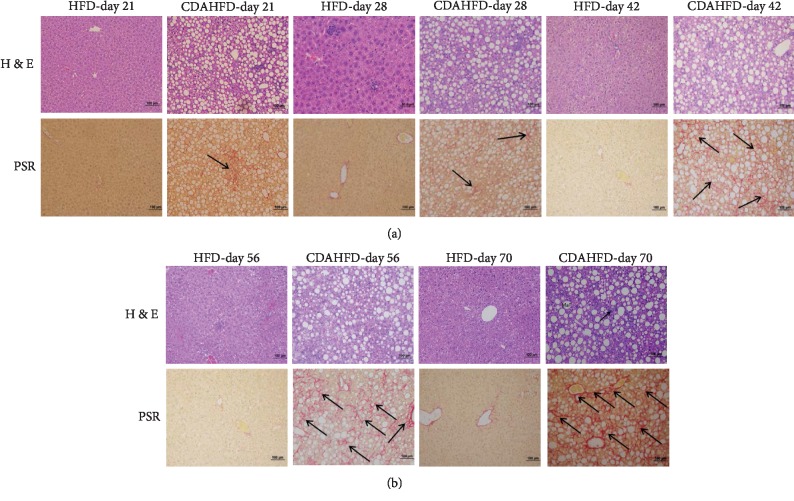
Hepatic histopathology of mice fed with CDAHFD or HFD from day 21 until day 70: (a) H&E staining and (b) Picro Sirius Red staining (PSR). All images represented at 200x.

CDAHFD or HFD livers were evaluated microscopically for the histological presence of hepatocellular inflammation, fat changes (steatosis), ballooning, and fibrosis. CDAHFD-fed mice showed a clear progression of NASH from day 21 with micro and macrovesicular fat changes observed from day 21 which progressed steadily until day 70. Grade 1 hepatic ballooning was observed from day 21 to 56 and grade 2 ballooning from day 56 until the end of the study. These changes were accompanied by grade 1 inflammatory foci from day 21 and grade 2 from day 35 onwards. Fibrosis was detected early in CDAHFD animals with stage 1 on day 21 progressing to stage 2 by day 35 and grade 3 from day 56 onward. In contrast, HFD-fed mice developed some steatosis and inflammatory foci at later time points but did not develop fibrosis over the time course studied (Figure 1).

### 3.2. PET Imaging of Integrin *α*_V_*β*_3_

**Figure 2:**
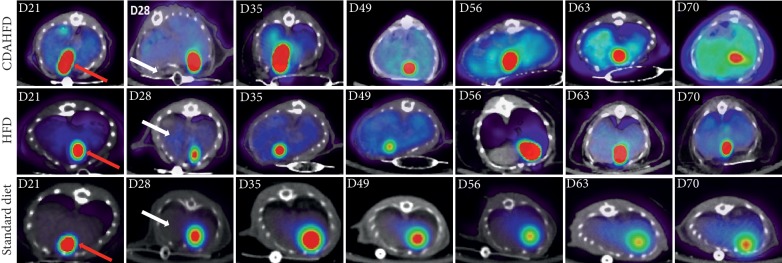
Representative images of [^18^F]FtRGD uptake in CDAHFD, HFD, and standard diet-fed mouse livers over the time course studied; red arrows indicate gall bladder uptake and white arrows depict the liver.

**Figure 3:**
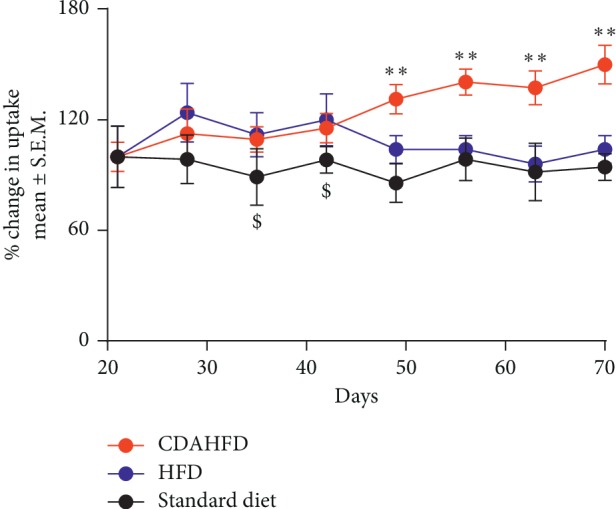
PET-derived [^18^F]FtRGD uptake in livers of CDAHFD, HFD, and standard diet-fed animals (*n* = 5). All animals were imaged 60 min after injection of [^18^F]FtRGD, and uptake was expressed as % change from day 21. Uptake of [^18^F]FtRGD in CDAHFD livers was significantly increased from day 49 (^*∗∗*^*p* < 0.01) compared with HFD-fed animals and from day 35 (^$^*p* < 0.05), when compared with mice fed with standard diet. Data are represented as mean ± SD.

Animals were imaged longitudinally for 10 weeks with [^18^F]FtRGD to determine how early imaging can detect the development of fibrosis. No significant difference in liver retention of [^18^F]FtRGD was observed at early time points between the CDAHFD fibrogenic diet-treated animals and either the HFD or standard diet fed mice as detailed in Table 2. Significant increases in liver retention of [^18^F]FtRGD were only observable in CDAHFD animals from day 35 when compared with standard diet-fed animals (*p* < 0.05) and significantly increased in the CDAHFD-fed mice compared with HFD mice from day 49 (*p* < 0.001), as shown in Figures 2 and 3.

### 3.3. Immunohistochemical Analysis

**Figure 4:**
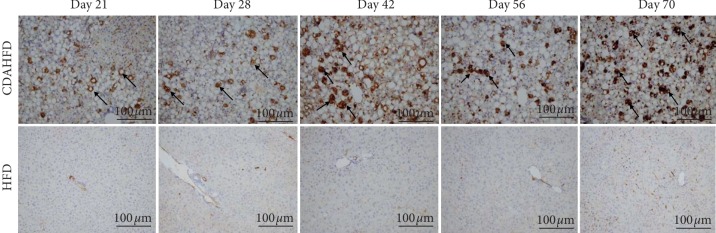
Representative photomicrographs showing *α*-SMA immunohistochemistry in liver tissue of CDAHFD- or HFD-fed mice. Upper panels show data from CDAHFD animals, and lower panels show data from HFD animals; black arrows represent areas of *α*-SMA staining.

Immunohistochemical assessment of smooth muscle actin (*α*-SMA) protein expression was used to measure the activation of hepatic stellate cells. Scoring was assigned for each liver based on the intensity of IHC staining. The expression of *α*-SMA slowly increased from day 21 to 70 and showed similar expression in all liver lobes. At day 21, the expression was minimal (<25%), by day 28 the expression was mild (25–50%), and reached moderate levels by day 42 (50–75%). On day 70, the expression of *α*-SMA was intense and covered most of the liver parenchyma (>75%). In contrast, HFD fed mice showed minimal expression of *α*-SMA around the hepatic artery/vein from day 21 to 56, only increasing to mild by day 70 (Figure 4).

### 3.4. Hepatic Expression of Genes Associated with Liver Fibrosis

**Figure 5:**
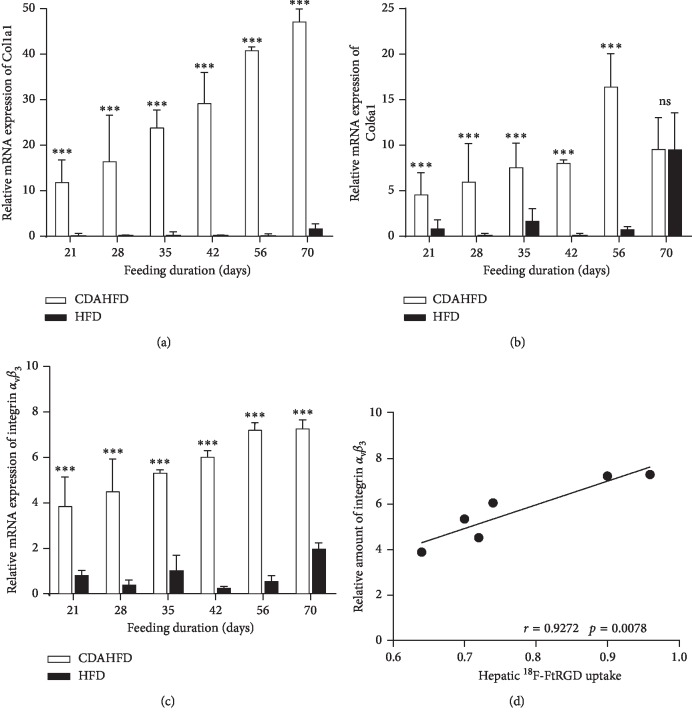
Relative mRNA expression of genes associated with fibrosis in CDAHFD- and HFD-fed mice. (a) Collagen 1a1, (b) collagen 6a1, and (c) integrin *α*_V_*β*_3_ were increased in CDAHFD-fed liver compared with HFD-fed mice from day 21. Data are expressed as fold difference, mean ± SD (^*∗∗∗*^*p* < 0.001, ns: no significant). (d) Correlation between hepatic uptake of [^18^F]FtRGD and mRNA expression of integrin *α*_V_*β*_3_ (Pearson's correlation: *r* = 0.9272, *p*=0.0078).

Hepatic mRNA expression levels of integrin *α*_V_*β*_3,_ collagen 1a, and collagen 6a were measured using qPCR to assess the progression of fibrosis. As shown in Figure 5, mRNA levels of collagen 1a and collagen 6a were significantly higher (*p* < 0.001) in mice fed with CDAHFD when compared with HFD mice from day 21 onwards. Integrin *α*_V_*β*_3_ levels also significantly increased (*p* < 0.001) in CDAHFD mice from day 21 and showed an excellent correlation to hepatic uptake of [^18^F]FtRGD (Pearson *r* = 0.9272, *p*=0.0078, Figure 5(d)).

## 4. Discussion

In the current study, we have assessed the utility of [^18^F]FtRGD for the early detection of liver fibrosis in a diet-induced murine model of NASH. The CDAHFD-fed model was developed as a NASH mouse model with a clinically relevant onset and progression of hepatic fibrosis [12, 19]. Alternative diet-induced models (such as the high fructose or combination high fat-high fructose diet) have been shown to develop mild levels of fibrosis, while chemically induced models (such as carbon tetrachloride or thioacetamide or cycloheximide models) develop severe fibrosis, neither of which mimics clinical pathology. Furthermore, these models do not develop the characteristic fat, triglycerides, and cholesterol deposits associated with the NASH liver [12, 14, 19, 20].

The progression of NASH pathology in the CDAHFD model in our study was confirmed using histological and biochemical measures and correlated to hepatic mRNA expression of collagen (col1a and col6a) and integrin *α*_V_*β*_3_. The histology data clearly shows the development of liver fibrosis in CDAHFD-fed animals with grade 1 fibrosis observed on day 21. By day 35, fibrosis had progressed to stage 2 and both serum triglycerides and hydroxyproline levels were elevated, along with significant increases in mRNA levels of collagen type 1 and integrin *α*_V_*β*_3_. A previous study by Rokugawa et al. assessed [^18^F]FPP-RGD_2_, a cyclic RGD peptide, in the CDAHFD model compared with standard diet alone and showed increased [^18^F]FPP-RGD_2_ retention in the liver but did not correlate this to fibrosis stage [8]. In the current study, we have longitudinally correlated [^18^F]FtRGD uptake and fibrosis every 7 days and compared uptake with both standard diet and an HFD which more accurately models the obesity, insulin resistance, and hyperlipidemia often associated with NASH clinically [1]. [^18^F]FtRGD uptake was significantly increased in CDAHFD animals compared with HFD from day 49 onwards and was strongly correlated with hepatic mRNA expression of integrin *α*_V_*β*_3_ (Pearson = 0.9272, *p*=0.0078). At day 49, the CDAHFD-fed liver showed evidence of extensive stage 2 fibrosis that progressed to stage 3 by day 56. When compared with standard diet-fed animals, [^18^F]FtRGD uptake was significantly increased in CDAHFD animals from day 35, and at day 35, the CDAHFD-fed liver showed early evidence of mild stage 2 fibrosis. These data suggest that the choice of control used to assess fibrosis is important and that the pathology associated with fatty liver can affect [^18^F]FtRGD uptake in the absence of fibrosis. The most sensitive conventional noninvasive imaging technique used clinically at present is MR-elastography which measures liver stiffness reliably and allows assessment of liver fibrosis at early stage 2 [3, 13, 21, 22] similar to [^18^F]FtRGD when compared with standard diet; new diagnostic imaging agents would be needed to improve the sensitivity and temporal assessment of fibrosis to change clinical practice.

## 5. Conclusions

In this study, we have assessed the utility of the radiopharmaceutical [^18^F]FtRGD for the early detection of liver fibrosis in a clinically relevant diet-induced NASH model. Histology confirmed the progression of liver fibrosis in the CDAHFD model and correlated strongly with mRNA expression of genes associated with fibrosis. Liver uptake of [^18^F]FtRGD was significantly increased in CDAHFD animals compared to HFD and SD animals and was strongly correlated with hepatic mRNA expression of integrin *α*_V_*β*_3_. Overall PET imaging with [^18^F]FtRGD showed similar detection sensitivity to the current clinical gold standard modalities for staging of liver fibrosis and provides further support for the development of integrin-based radiopharmaceuticals for the assessment of fibrosis in NASH.

## Figures and Tables

**Table 1:** The physiological measures of liver disease in mice fed with CDAHDF (*n* = 5) compared with HFD-fed mice (*n* = 5). All data are presented as mean ± SD, and statistical significance is shown as ^*∗*^*p* < 0.05, ^*∗∗*^*p* < 0.01, and ^*∗∗∗*^*p* < 0.001.

Days	Liver weight (g)		Triglycerides (mg/g)		Hydroxyproline (*μ*g/g)	
	CDAHFD	HFD	CDAHFD	HFD	CDAHFD	HFD
21	1.5 ± 0.05^*∗*^	1.15 ± 0.02	43.86 ± 2.17	47.34 ± 2.17	4.29 ± 0.17	4.71 ± 0.47
28	1.69 ± 0.02^*∗*^	1.27 ± 0.05	49.46 ± 2.39	45.94 ± 1.82	4.95 ± 0.54	4.58 ± 0.39
35	1.85 ± 0.17^*∗∗*^	1.30 ± 0.05	56.83 ± 1.50^*∗*^	42.87 ± 3.67	5.68 ± 0.14^*∗*^	4.25 ± 0.62
42	2.00 ± 0.11^*∗∗∗*^	1.40 ± 0.06	59.67 ± 1.52^*∗∗*^	41.53 ± 2.50	5.96 ± 0.16^*∗∗*^	4.15 ± 0.42
56	2.20 ± 0.06^*∗∗∗*^	1.46 ± 0.08	61.47 ± 1.18^*∗∗∗*^	40.03 ± 1.50	6.17 ± 0.12^*∗∗*^	4.04 ± 0.24
70	2.23 ± 0.18^*∗∗∗*^	1.51 ± 0.10	63.34 ± 2.67^*∗∗∗*^	37.16 ± 0.88	6.35 ± 0.26^*∗∗∗*^	3.53 ± 0.13

**Table 2:** Liver uptake of [^18^F]FtRGD in mice fed with CDAHDF, HFD, and standard diet-fed mice (*n* = 5 per group). All data are presented as mean ± SD, and statistical significance is shown as ^*∗*^*p* < 0.05, ^*∗∗*^*p* < 0.01, and ^*∗∗∗*^*p* < 0.001.

Days	Standard diet %ID/g ± SD	High fat diet %ID/g ± SD	CDAHFD %ID/g ± SD
21	0.52 ± 0.13	0.50 ± 0.19	0.64 ± 0.11
28	0.54 ± 0.16	0.62 ± 0.18	0.72 ± 0.19
35	0.46 ± 0.11$	0.56 ± 0.13	0.70 ± 0.10
42	0.57 ± 0.09$	0.60 ± 0.16	0.74 ± 0.11
49	0.47 ± 0.11$$	0.52 ± 0.08	0.84 ± 0.11^*∗∗*^
56	0.53 ± 0.09$$	0.52 ± 0.08	0.90 ± 0.10
63	0.48 ± 0.14$$	0.48 ± 0.11	0.88 ± 0.13^*∗∗*^
72	0.57 ± 0.10$$	0.51 ± 0.09	0.96 ± 0.15

## Data Availability

The datasets used and/or analyzed during the current study are available from the corresponding author on reasonable request.
